# Socioeconomic inequalities in breast and cervical screening coverage in England: are we closing the gap?

**DOI:** 10.1177/0969141315600192

**Published:** 2015-09-16

**Authors:** Elaine Douglas, Jo Waller, Stephen W Duffy, Jane Wardle

**Affiliations:** 1 Health Behaviour Research Centre, Department of Epidemiology & Public Health, University College London, Gower Street, London, WC1E 6BT; 2 Centre for Cancer Prevention, Wolfson Institute of Preventive Medicine, Queen Mary University of London, London, EC1M 6BQ

**Keywords:** cancer screening, coverage, socioeconomic inequalities

## Abstract

**Objective:**

Health policy in the UK is committed to tackling inequalities in cancer screening participation. We examined whether socioeconomic inequalities in breast and cervical cancer screening participation in England have reduced over five years.

**Methods:**

Cross-sectional analyses compared cervical and breast screening coverage between 2007/8 and 2012/13 in Primary Care Trusts (PCTs) in England in relation to area-level income deprivation.

**Results:**

At the start and the end of this five year period, there were socioeconomic inequalities in screening coverage for breast and cervical screening. Inequalities were highest for breast screening. Over time, the coverage gap between the highest and lowest quintiles of income deprivation significantly reduced for breast screening (from 12.3 to 8.3 percentage points), but not for cervical screening (5.3 to 4.9 percentage points).

**Conclusions:**

Efforts to reduce screening inequalities appear to have resulted in a significant improvement in equitable delivery of breast screening, although not of cervical screening. More work is needed to understand the differences, and see whether broader lessons can be learned from the reduction of inequalities in breast screening participation.

## Introduction

The National Health Service (NHS) Breast and Cervical Screening Programmes were introduced in the UK in 1988. In England, breast screening is offered every three years to women aged 50–70, with an age-extension from 47–73 years currently being rolled out.^1^ In the face of recent debate about the value of the breast screening, an independent review was carried out in 2012, which concluded that, on balance, screening is beneficial.^2^ Cervical screening is offered to women aged 25–64, every three years for women aged 25–49 and every five years for those aged 50–64.^3^ Both programmes use a ‘call-recall’ system in which women receive invitations, re-invitations, and reminders, as recommended by the World Health Organization (http://www.who.int/cancer/detection/variouscancer/en/).

Coverage (defined as breast screening within the past three years, and cervical screening within the past five years) is high for both programmes: currently 77% for breast screening^4^ and 78% for cervical screening,^3^ although cervical screening coverage has been declining, particularly in younger women,^3^ and has not reached its 80% target since 2005. There is also long-standing concern that coverage across both programmes is lower among women from lower socioeconomic status (SES) backgrounds. Using area-level measures of SES, breast and cervical screening coverage has been found to be lower in more deprived areas.^5–9^ Using individual-level measures, women who live in rented accommodation or in households without cars have been shown to be significantly less likely to attend breast screening.^10^ Educational level has also been associated with lower cervical screening coverage in a number of national surveys.^10–13^ Inequalities in coverage of breast and cervical screening are likely to be contributing to inequalities in cancer outcomes.^14,15^

Successive UK governments have made policy commitments to tackling inequalities in cancer screening participation.^1,16^ Building on this commitment, there have been many local activities designed to promote screening coverage in deprived areas. These have adopted a variety of strategies, including GP endorsement, addressing programme-specific barriers, and developing socially and culturally appropriate invitation approaches.^17,18^ Cumulatively, these may have contributed to a reduction in socioeconomic inequalities in screening coverage over time.

Until 2013, Primary Care Trusts (PCTs) were responsible for screening coverage within their areas. National, quality-assured PCT-level coverage data are available for download from the NHS Health and Social Care Centre and are not subject to self-report bias inherent in individual-level data. The present study therefore examined associations between area-level deprivation and breast and cervical screening coverage in England from 2007 to 2012.

## Methods

PCT^1^ data on breast and cervical screening coverage for the period 2007–2012 were downloaded from the Health and Social Care Information Centre.^4,19^ We included data from all 151 PCTs in England, 31 of which were in London.

Breast screening coverage data were for women aged 53–70. Breast screening coverage is defined as the percentage of eligible women who have had a test with a recorded result in the last three years.^4^ Cervical screening data were available for women aged 25–64, but we also subdivided the sample into those aged 25–49 (using 3.5 year coverage) and 50–64 (using five year coverage), for age-matched comparison with breast screening. Cervical screening coverage is defined as the percentage of eligible women who have had a test with a recorded result in the last 3.5 years for those aged 25–49 and in the last five years for those aged 50–64.^4^

We used the income domain score from the Index of Multiple Deprivation (IMD) 2010 as the marker of deprivation. This is an area-level measure based on the number of households on low income, benefits or other welfare support. The score is the proportion of people classed as income deprived, and is calculated using a population-weighted average of Lower Super Output Area income deprivation score, aggregated to PCT level. IMD scores at PCT level were downloaded from the National Gynaecological Hub.^20^ IMD scores were categorized into quintiles for the primary analyses.

### Statistical analysis

Data were analysed using Stata version 10.1.^21^ Descriptive statistics were generated for PCT-level coverage of both screening programmes. To describe the relationship between screening coverage and income deprivation, we fitted a Poisson regression model by quintiles of IMD. We examined changes over time by testing for an interaction between time and income deprivation in their combined effect on coverage in the Poisson regression model. This is equivalent to estimating and testing the effect of the product of year and IMD on coverage.

## Results

### Screening Coverage

Annual coverage figures for the two programmes from 2007 to 2012 are shown in Table 1. Overall, breast screening coverage was fairly stable at 74–75%, although the range shows that there was an improvement in the worst-performing PCTs, with the minimum coverage increasing from 43.9% in 2007/8 to 58.3% in 2012/13. Overall cervical screening coverage was also stable at around 78%, with little change in the range across PCTs.

**Table 1.** Descriptive statistics for breast and cervical screening coverage within PCTs, England (2007–2012).

	Breast screening coverage (%)			Cervical screening coverage (%)		
Year	Min-Max	Mean	SD	Min-Max	Mean	SD
2007–08	43.9–84.6	74.6	8.1	66.7–85.7	78.1	3.7
2008–09	50.9–84.8	75.1	7.6	65.8–85.8	78.5	3.8
2009–10	56.9–85.0	75.6	6.2	66.4–85.4	78.5	3.6
2010–11	59.8–85.1	75.9	5.3	67.2–84.3	78.3	3.4
2011–12	59.5–84.7	75.6	5.1	65.9–83.8	78.3	3.4
2012–13	58.3–83.3	74.8	5.3	65.5–83.5	78.0	3.4

### Deprivation and Screening Coverage

**Figure 1.**
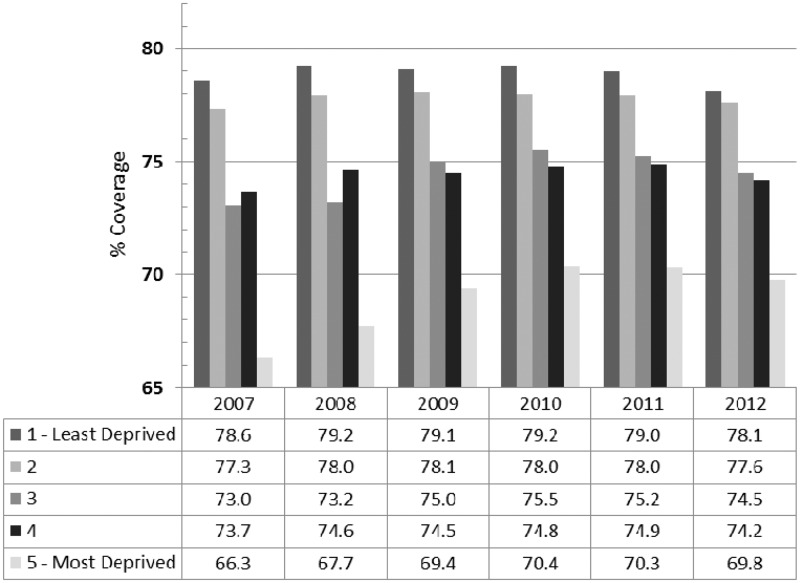
Breast screening coverage by quintile of area-level deprivation within PCTs, England, age 53–70 (2007–12).

Figure 1 shows breast screening coverage by PCT-level quintile of income deprivation across the time period of the study. In 2007, the difference in coverage between the least deprived (Q1) and most deprived (Q5) quintile was 12.3 percentage points. Coverage in the less deprived quintiles changed little, but coverage in Q5 increased from 66.3% to 69.8%, suggesting that inequalities improved over time. Table 2 shows the relative rates of coverage from the Poisson regression. Poisson regression models for each year showed that Q3, Q4 and Q5 had significantly lower coverage than Q1 (see Table 2). However, the relative coverage for Q5 was 0.85 (95% CI 0.84–0.86) in 2007/8 and increased to 0.89 (95% CI 0.88–0.90) in 2012/13. There was significant heterogeneity in the association of coverage with income deprivation by year (p < 0.0001), with the strength of the negative association declining significantly with successive years.

**Table 2.** Results of Poisson regression of breast screening coverage on quintiles of deprivation by year, England (2007–12).

Year	Deprivation quintile	RR*	95% CIs		Average % coverage	p
			Lower	Upper		
2007–08	Q1 (reference)	1.00			79	–
	Q2	0.99	0.98	1.00	77	<.0001
	Q3	0.94	0.93	0.95	73	<.0001
	Q4	0.94	0.93	0.95	74	<.0001
	Q5 (most deprived)	0.85	0.84	0.86	66	<.0001
2008–09	Q1 (reference)	1.00			79	–
	Q2	−.99	0.98	1.00	78	<.0001
	Q3	0.93	0.92	0.94	73	<.0001
	Q4	0.95	0.94	0.96	75	<.0001
	Q5 (most deprived)	0.86	0.85	0.87	68	<.0001
2009–10	Q1 (reference)	1.00			79	–
	Q2	1.00	0.99	1.01	78	.2
	Q3	0.95	0.94	0.96	75	<.0001
	Q4	0.95	0.94	0.96	75	<.0001
	Q5 (most deprived)	0.88	0.87	0.89	70	<.0001
2010–11	Q1 (reference)	1.00			79	–
	Q2	0.99	0.98	1.00	78	<.0001
	Q3	0.96	0.95	0.97	76	<.0001
	Q4	0.95	0.94	0.96	75	<.0001
	Q5 (most deprived)	0.89	0.88	0.90	70	<.0001
2011–12	Q1 (reference)	1.00			79	–
	Q2	0.99	0.98	1.00	78	.01
	Q3	0.95	0.94	0.96	75	<.0001
	Q4	0.95	0.94	0.96	75	<.0001
	Q5 (most deprived)	0.89	0.88	0.90	70	<.0001
2012–13	Q1 (reference)	1.00			78	–
	Q2	1.00	0.99	1.01	78	.6
	Q3	0.96	0.95	0.97	75	<.0001
	Q4	0.95	0.94	0.96	74	<.0001
	Q5 (most deprived)	0.89	0.88	0.90	70	<.0001

* Relative rate of coverage.

**Figure 2.**
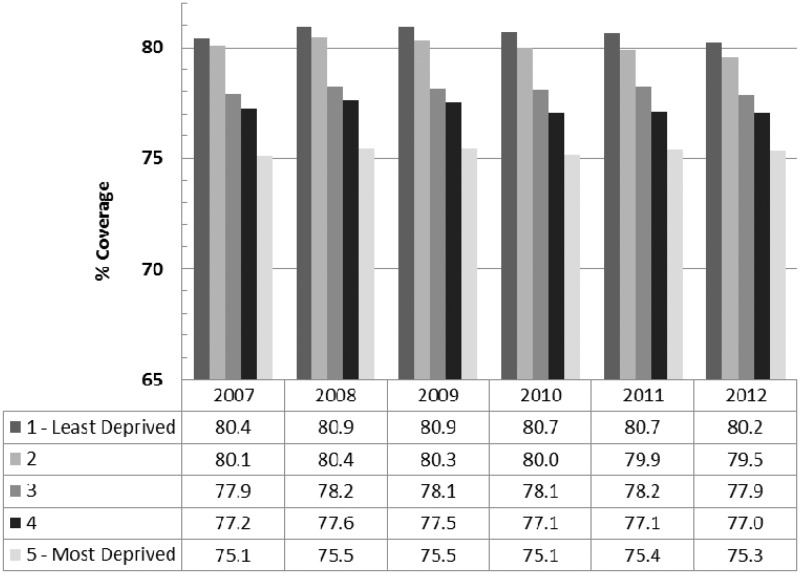
Cervical screening coverage by quintile of deprivation within PCTs, England, age 25–64 (2007–12).

Figure 2 shows cervical screening coverage for women aged 25–64 by quintile of income deprivation. In 2007, the difference in coverage between Q1 and Q5 was 5.3 percentage points, considerably smaller than the breast screening coverage gap. Unlike the pattern for breast screening, coverage was fairly stable across time in all quintiles, with little evidence of a reduction in the socioeconomic gradient, although there was a relatively high coverage in the least deprived quintile in 2009–10, yielding a stronger gradient for that year. As with breast screening, Poisson regression analyses showed that Q3, Q4 and Q5 (and in most years Q2) had significantly lower cervical screening coverage than Q1 (Table 3). The coverage gradient ran from 80% (least deprived) to 75% (most deprived) in both 2007–08 and 2012–13. There was significant heterogeneity in the relationship between coverage and income deprivation across years (p < 0.0001). This was not due to a decline in the gradient over time, but rather a peak in the gradient in year 2009–10.

**Table 3.** Results of Poisson regression of cervical screening coverage on quintiles of deprivation by year, England (2007–12).

Year	Deprivation quintile	RR*	95% CIs		Average % coverage	p
			Lower	Upper		
2007–08	Q1 (reference)	1.00			80	–
	Q2	1.00	0.99	1.01	80	.4
	Q3	0.96	0.95	0.97	78	<.0001
	Q4	0.95	0.94	0.96	77	<.0001
	Q5 (most deprived)	0.93	0.92	0.94	75	<.0001
2008–09	Q1 (reference)	1.00			81	–
	Q2	0.99	0.98	1.00	80	.008
	Q3	0.96	0.95	0.97	78	<.0001
	Q4	0.95	0.94	0.96	78	<.0001
	Q5 (most deprived)	0.93	0.92	0.94	76	<.0001
2009–10	Q1 (reference)	1.00			84	–
	Q2	0.93	0.92	0.94	80	<.0001
	Q3	0.89	0.88	0.90	78	<.0001
	Q4	0.88	0.87	0.89	77	<.0001
	Q5 (most deprived)	0.86	0.85	0.87	76	<.0001
2010–11	Q1 (reference)	1.00			81	–
	Q2	0.99	0.98	1.00	80	<.0001
	Q3	0.96	0.95	0.97	78	<.0001
	Q4	0.95	0.94	0.96	77	<.0001
	Q5 (most deprived)	0.93	0.92	0.94	75	<.0001
2011–12	Q1 (reference)	1.00			81	–
	Q2	0.99	0.98	1.00	80	<.0001
	Q3	0.97	0.96	0.98	78	<.0001
	Q4	0.95	0.94	0.96	77	<.0001
	Q5 (most deprived)	0.93	0.92	0.94	76	<.0001
2012–13	Q1 (reference)	1.00			80	–
	Q2	0.99	0.98	1.00	80	<.0001
	Q3	0.97	0.96	0.98	78	<.0001
	Q4	0.95	0.94	0.96	77	<.0001
	Q5 (most deprived)	0.94	0.93	0.95	75	<.0001

* Relative rate of coverage.

**Figure 3.**
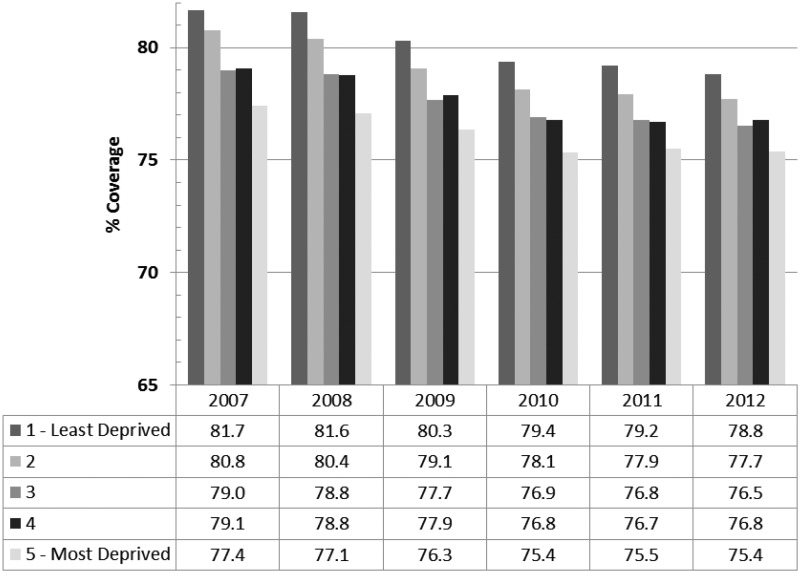
Cervical screening coverage by quintile of deprivation within PCTs, England, in women aged 50–64 (2007–12).

We repeated the cervical screening analyses restricted to women aged 50–64 (five year coverage data), to be age-comparable with the breast screening programme. The pattern of findings was very similar to the full age distribution. Figure 3 shows that the pattern of coverage across quintiles is much more similar to the pattern for cervical screening across all ages than the pattern of breast screening. This suggests that programmatic differences underlie the different patterns of association, rather than the age of the women invited.

## Discussion

Associations between income deprivation and screening coverage are well documented,^7,12,18^ but few studies have examined whether inequalities are changing. We found, as expected, that PCTs with higher levels of income deprivation had lower coverage for both breast and cervical screening. However, for breast screening, the difference in coverage between the most and least deprived PCTs narrowed significantly over the five years from 2007/8 to 2012/13. This was not the case for cervical screening, even when the analyses were restricted to women of an age comparable with those in the breast screening programme. This suggests that while strategies to increase breast screening coverage in poorer areas of England may have been successful, the low uptake of cervical screening in poorer areas has been more resistant to change, though the coverage gap is still greater for breast than cervical screening. In 2012/13 the difference in coverage between the highest and lowest quintiles of income deprivation was 8.3 percentage points for breast screening and 4.9 percentage points for cervical, so there is still scope for improvement.

Differences in screening coverage across the programmes may be due to the particular characteristics of each screening programme.^22^ For example, women invited to cervical screening are asked to arrange an appointment for screening at their own General Practice, whereas women invited to breast screening are provided with a scheduled appointment at a breast screening unit, generally in a local hospital or mobile unit. However, as yet it is unclear whether these factors might have a differential impact on women from different socioeconomic backgrounds and therefore what their contribution to the SES gradients in coverage might be.

There has been a suggestion that lower screening uptake – regardless of the characteristics of the unscreened group – should be respected as the result of an informed choice.^23^ The evidence suggests otherwise. At least in the case of colorectal screening, the unscreened group is much less likely to read the information provided with the screening invitation.^24^ This suggests that they are more likely to be unengaged than making an informed choice, particularly as their health literacy tends to be lower.^25^ In addition, even in countries such as the UK, where medical care is delivered without cost to the individual, many barriers to screening across programmes - social, fear of the test, embarrassment - are more prevalent in more deprived groups.^26^ Until we ensure that information and access are socially equitable, it is not appropriate to interpret uptake differences as a consequence of an informed choice.

One unexpected finding was the peak in cervical screening coverage in 2009–10. This may have been related to death from cervical cancer of the television celebrity, Jade Goody,^27^ though we are not aware of previous work which suggests that the impact was strongest among less deprived groups.

### Strengths and Limitations

This study benefited from complete data on uptake as a result of using routinely collected data, so there were none of the problems associated with differential response rates in more deprived population subgroups. Methods of data collection over time were also the same, making it possible to interpret differences. But there were also limitations. Area-based measures of deprivation do not reflect the granularity of variation between individuals, and may therefore show a different relationship with behaviour.^28^ However, this is the only option when using routinely collected data without individual permissions to access screening records. In this study they were based on scores at Lower Super Output Areas, which are relatively small, homogenized geographic units, and then weighted to PCT level, so they should be fairly accurate. In addition, we only used the income domain because the full IMD includes the health domain and may incur a ‘mathematical coupling’.^29^

## Conclusion

A reduction in the breast screening coverage gap across English PCTs suggests that efforts to reduce screening inequalities may have had an effect. However, for cervical screening, there has been no discernible improvement in inequalities, although the magnitude of the inequality effect was consistently lower for cervical than breast screening. More work is needed to understand the differences, and to see what lessons can be learned from the reduction of inequalities in breast screening participation to apply to other programmes, such as colorectal screening, which have very high inequalities.

## Footnotes

1 Primary Care Trusts (PCTs) were local NHS healthcare organisations which, until March 2013, were responsible for cancer screening in their local area. The median population covered by a PCT was 284,000 http://www.kingsfund.org.uk/blog/2012/07/ccgs-and-pcts-not-so-different-after-all
